# The Impact of Different Closures on the Flavor Composition of Wines during Bottle Aging

**DOI:** 10.3390/foods10092070

**Published:** 2021-09-02

**Authors:** Isabel Furtado, Paulo Lopes, Ana Sofia Oliveira, Filipa Amaro, Maria de Lourdes Bastos, Miguel Cabral, Paula Guedes de Pinho, Joana Pinto

**Affiliations:** 1 Associate Laboratory i4HB—Institute for Health and Bioeconomy, Laboratory of Toxicology, Department of Biological Sciences, Faculty of Pharmacy, University of Porto, Rua Jorge Viterbo Ferreira, 228, 4050-313 Porto, Portugal; isabel.c.furtado95@gmail.com (I.F.); aoliveira@ff.up.pt (A.S.O.); famaro@ff.up.pt (F.A.); mlbastos@ff.up.pt (M.d.L.B.); pguedes@ff.up.pt (P.G.d.P.); 2 UCIBIO/REQUIMTE, Laboratory of Toxicology, Department of Biological Sciences, Faculty of Pharmacy, University of Porto, Rua Jorge Viterbo Ferreira, 228, 4050-313 Porto, Portugal; 3 Amorim Cork, S.A., Rua dos Corticeiros, 850, 4536-904 Santa Maria de Lamas, Portugal; paulo.lopes@amorim.com (P.L.); miguel.cabral@amorim.com (M.C.)

**Keywords:** wine bottle closures, volatile composition, wine flavor, desorption, scalping

## Abstract

Wine flavor undergoes major changes during bottle aging and can be influenced by the type of closure. The interaction between wine, the type of closure and the external environment has the potential to significantly influence the overall quality of bottled wines, especially when the storage period is relatively long (more than five years). Therefore, the choice of closure (cork, synthetic or screw cap) deserves special attention in order to establish the ideal sealing conditions for optimizing wine flavor attributes. The contribution of different closures to the quality of bottled wine is through mass transfer phenomena, including permeation, sorption (scalping) or desorption of chemicals between closure materials and wines. Thus, this article aims to review the impact of different closures on the flavor composition of wines during post-bottling conditions. The implications of closures on wine sensory properties are also discussed.

## 1. Introduction

Wine flavor is one of the most important factors in establishing wine quality and consumer acceptance. The perception of wine flavor and aroma is the result of a multitude of interactions between odor-active molecules (volatile organic compounds) and human sensory receptors [1]. Esters, higher alcohols, aldehydes, fatty acids, terpenes, and sulfur and volatile phenolic compounds are some examples of odor-active molecules that most contribute to wine flavor. The final sensory quality of a wine varies due to the combination of different factors, such as the grape cultivar, geographic origin, vinification production and technological processes, and post-bottling conditions [1,2].

In particular, post-bottling conditions can lead to the development of different wine characteristics due to different storage conditions (temperature, light, humidity, and bottle position in cellar) as well as different packaging and sealing materials [3,4,5]. Among these, the closure type has been considered one of the most determinant factors in the process of wine aging. Bottle closures highly affect the organoleptic properties of bottled wines, since they can influence oxygen permeation and promote the desorption of several volatile compounds into wine, which can contribute to wine’s flavor. In addition, closures display sorption behaviors for several wine compounds, resulting in a decrease in wine quality and its shelf life by altering the aroma compound profile.

The market for wine closures is currently dominated by three main bottle-closure technologies: cork, synthetic, and screw caps. Each closure type has its own features, advantages, and limitations; this review is focused on the impact of these closures on wine flavor composition during bottle aging.

## 2. Types of Wine Bottle Closures

### 2.1. Cork Stoppers

Cork is the bark of *Quercus suber* L., which exists mainly in the western Mediterranean, specifically in Portugal, Spain, Italy, France, Morocco, Tunisia, and Algeria [6]. Cork has been used over the centuries as a bottle closure due to its physical properties, namely high flexibility, elasticity, compressibility, and recovery as well as very low permeability to liquids and low density [7]. These characteristics are the result of cork cellular structure and composition, i.e., the highly organized arrangement of small, hollow, and closed cells, usually referred as honeycomb, with suberin and lignin as the main components of the cell’s wall [7,8].

The cork is manually extracted during the spring and summer seasons, mainly in cycles of nine years, depending on the geographical region, reaching an increase of 3–3.5 cm in the cork plank’s thickness [9]. After extraction, cork goes through several stages of the manufacturing process, depending on the type of stopper to be produced.

Cork stoppers include natural and technical corks. Natural stoppers are produced from planks that are cut into strips and perforated with a drill [10]. Then, the stoppers are washed, disinfected, bleached with hydrogen peroxide, and graded according to their level of porosity. Finally, they are branded (using food-quality ink or heat or laser marking), lubricated with silicon or paraffin, and packaged [9].

Technical cork stoppers are the result of natural cork production by-products converted into granules, which are conglomerated using a Food and Drug Administration (FDA)-approved binder (e.g., polyurethane glue) [11]. This class includes agglomerated cork stoppers with larger granules (2–9 mm), microagglomerated cork stoppers with smaller granules (0.5–2 mm), and stoppers composed of a densely agglomerated cork body, with two discs of natural cork glued in one (“2 + 0” technical stopper) or both ends (“1 + 1” technical stopper) [9]. These discs of natural cork are obtained from the punching of thin planks that do not have adequate thickness to produce natural cork stoppers and that are bonded to the agglomerated body using an FDA-approved binder [9].

### 2.2. Synthetic Closures

Synthetic closures appeared on the market in the mid-1990s to overcome the presence of 2,4,6-trichloroanisole (TCA) in cork stoppers, which is produced by fungi and can be desorbed into the wine, leading to *musty* and *moldy* off-flavors [3,12]. This type of closure can be produced through two different methods, namely polymer injection and co-extrusion [13]. The first method consists of using thermoplastic elastomer mixtures (styrene-butadiene-styrene and styrene-ethylene/butadiene-styrene) injected into a mold cavity [13]. On the other hand, the co-extrusion process occurs in two main stages: (1) the raw materials (low density polyethylene (LDPE) and talc) are combined, melted and then extruded in order to create a long foamed cylinder, which is the core of the closures; and (2) the closure’s core is thermally bonded with an outer flexible skin (LDPE-based thermoplastic elastomer) by an extrusion process [13,14]. Synthetic closures are designed to try to mimic cork stoppers to meet consumer demands and are microbiologically inert [12]. However, some problems have emerged, such as the difficulty in removing the stopper from the bottle, high permeability to oxygen, scalping volatiles from wine, and promoting the desorption of some compounds into the wine, as will be further discussed below.

### 2.3. Screw Caps

Screw caps are metal caps that screw onto threads on the neck of the wine bottle, creating an airtight seal around the outside of the bottle neck [12,15]. The inner part of the screw cap is generally composed of a polyvinylidene chloride (PVDC) film in contact with the wine, a layer of tin foil acting as gas barrier, and a polyethylene (PE) wad to maintain compression. The most common liners in screw caps are Saran-tin and Saranex [15]. The Saran-tin liner is composed of successive layers of PE, Kraft paper, tin, and PVDC, while Saranex is composed of PE covered on both sides with PVDC.

Screw cap closures are easy to remove from the wine bottle and are considered a good choice of closure when the use of a corkscrew is undesirable [10]. However, the metal cap, usually aluminum, can lead to the releasing of metal ions into the wine during bottle aging, since it is not an inert material [10,16].

## 3. Contribution of Different Closures to Wine Flavor Composition during Aging

### 3.1. Oxygen Transmission Rate (OTR) of Closures

During aging, the oxygen ingress through the bottle is highly dependent on the sealing effectiveness of the closures, which present different oxygen barrier properties [17]. This is especially relevant for the wine industry, as wine oxidation over time and its shelf life are driven by the oxygen transfer of different wine closures. The methods used in the measurement of the oxygen barrier properties on the food packaging are very well established and described [18]. In contrast, the measurement of oxygen barrier properties of wine closures has required the development of specific methods, given their specific properties, considerable thickness, and the amount of oxygen trapped inside each closure. Over the years, different techniques have been proposed to measure oxygen ingress through different wine closures.

Lopes et al. (2005) optimized a non-destructive colorimetric method to measure oxygen ingress into wine bottles [19,20,21]. This method measures the oxygen ingress through closures by direct colorimetric scanning of colorless wine bottles containing reduced indigo carmine solutions, which gradually changes color from yellow to indigo as oxygen reacts progressively with the reduced indigo carmine. This method allows the measurement of the total oxygen that enters into a wine bottle over time, comprising the oxygen desorbed by the closures and the oxygen ingress rate through closures over time. This method presents the advantage of measuring the oxygen ingress through a fully compressed closure under real conditions during wine storage in bottles. However, the results of this method can be biased if the bottles are overexposed to the light and temperature [21].

Another non-invasive method for the measurement of oxygen ingress through different closures in wine bottles using chemiluminescence was developed [22]. This method determines the total oxygen entering into a wine bottle after closure insertion through a sensor dot containing a luminophore sensitive to oxygen, which is glued inside an empty transparent bottle. When the sensor is illuminated with an optical probe, it fluoresces and is then quenched by the oxygen. The reduction of fluorescence is then proportional to the partial pressure of oxygen inside the wine bottle. The concentration of oxygen inside the bottle is obtained using the ideal gas law. While this method has the advantage of being non-destructive, it uses empty bottles, i.e., dry conditions without the partial pressure of water and ethanol, which diverges from the real conditions of wine bottle aging. Under these conditions, closures with a more hydrophilic behavior, such as cork stoppers, are strongly penalized. Fonseca et al. (2013) reported that oxygen ingress through cork stoppers could be reduced by a factor of 10 when cork becomes soaked (wet conditions) (Table 1). To approximate the real conditions of wine bottle aging, several attempts have been made to determine the oxygen ingress through different closures by chemiluminescence, using bottles filled with wine as well as hydroalcoholic and acid solutions; however, several compounds present in the solution consume oxygen, leading to an underestimation of the oxygen ingress under these conditions [23,24].

Other methods can also be used to determine the oxygen ingress through the closure, although most of them consist of an indirect measure or are destructive, such as the coulometric and manometric methods. The coulometric method is based on the American Society for Testing and Materials (ASTM) standard F1927, which defines a procedure for “the determination of the rate of transmission of oxygen gas through film, sheeting, laminates, co-extrusions, or plastic coated papers or fabrics” [31]. This method consists of the measurement of the oxygen flow through closures inserted in a cut bottleneck under constant oxygen pressure applied on the outer closure surface while the inner part of the closure is flushed with nitrogen [25,26,32]. Permeation of oxygen through the closure is detected in the carrier gas outlet from the inner bottleneck under the closure, after steady state has been achieved, by electrochemical methods. The main drawback of this method is that closures are not tested under the real conditions of use in a wine bottle, i.e., in contact with aqueous ethanolic solutions. In addition, this method is used to determine OTR and does not consider the amount of oxygen released out of closures, which plays an important role in wine bottle aging.

A manometric method has also been used to establish the oxygen transfer through wine closures. This method consists of the measurement of pressure difference through outgassed cork laminates/stoppers in two closed chambers [33,34,35,36]. The oxygen transfer through the samples is monitored by the pressure decrease in the first chamber (initially submitted to 212 hPa or 1013 hPa) and the increase of pressure on the second chamber (initially submitted to dynamic or static vacuum conditions) over time. The oxygen transfer is then calculated based on Fick’s law applied to the steady state [37]. This method has the disadvantage of being carried out at 0% relative humidity on dry uncompressed samples. Moreover, OTR is obtained from measurements made on stopper laminates and is then extrapolated to the full cork. In the case of natural cork stoppers, which present a heterogenous structure, the extrapolation could lead to significant errors, as the full internal structure of cork and its arrangement are not taken into consideration. In addition, the measurements are conducted at steady state, which prevents the quantification of the oxygen that outgases from the closure when it is compressed into the bottleneck.

The resultant comparison is difficult due to the method conditions and the multiplicity of parameters and conditions involved (methods, units, time, storage, etc.) in the oxygen ingress determination. However, it seems that the colorimetric method is the approach that provides a more realistic measurement of the total oxygen that ingresses after closure insertion. This method is able to provide the most meaningful information to wine producers, namely the total oxygen ingress into a wine bottle after bottling, which includes the oxygen desorption from the closures and its OTR; either the oxygen passes through closure or between closure/glass interface.

**Figure 1:**
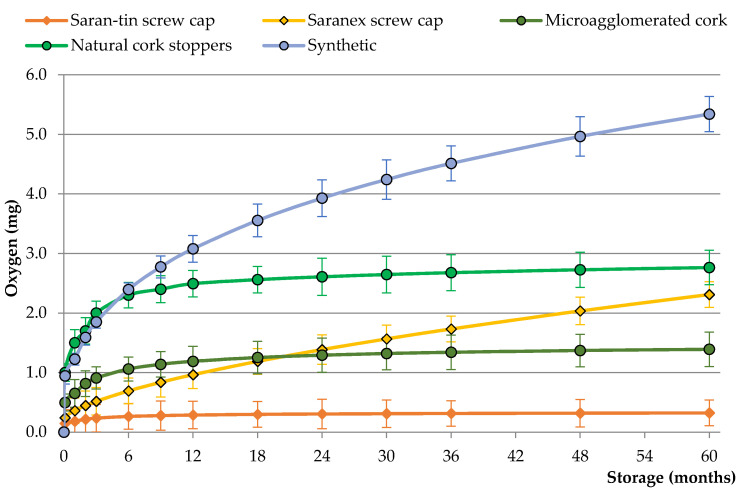
Oxygen ingress through different types of wine closures into bottles during five years of wine storage in a horizontal position.

The oxygen ingress into the wine bottle varies with the type of closure (Figure 1) [38]. Cylindrical closures inserted in the bottleneck, whether cork or synthetic, display distinctive kinetics. Wines sealed with these closures receive important amounts of oxygen during the first months, which comprises essentially oxygen released from the closures and oxygen inserted into the bottleneck by the closure piston effect during wine bottling. Following this period, which varies significantly depending on the type of closure, the oxygen ingress reaches a steady state, after which the OTR values can be calculated. The screw cap sealed bottles seem to reach the steady state immediately after bottling, while cylindrical closures can take from 6 to 12 months to reach a steady state, as can be seen in Figure 1.

Synthetic closures exhibit the highest oxygen ingress after bottling, mainly due to their very high OTR [17,37,39]. This oxygenation occurs throughout the PE foam that composes synthetic stoppers [17]. In contrast, screw caps display low OTR, depending on the type of liner used to seal the bottles [20,32]. The Saran-tin liner, composed of successive layers of PE, Kraft paper, tin or aluminum, and PVDC, is almost impermeable, exhibiting the lowest OTR values. However, the Saranex liner, composed of PE inserted between two PVDC layers, allows some level of oxygen ingress during wine storage in the horizontal position. In wines sealed with screw caps, the oxygen permeates through the liner or between the liner and glass, depending on how well the capsule was applied.

The barrier properties of cork stoppers present a better performance when compared with synthetic closures; however, oxygen ingress varies according to the type of cork stoppers (technical or natural) [3,19,20]. Thus, the oxygen ingress into bottles sealed with technical corks such as microagglomerate corks varies between 1.0 and 1.3 mg over time (Figure 1). Most of this oxygen diffuses out of the cork, due to the compression on the bottleneck, during the first six months of storage. After this period, the OTR throughout cork–glass interface or throughout the cork seems to be negligible, exhibiting values similar to the Saran-tin screw caps [17,35].

The natural cork stoppers exhibit similar oxygen ingress kinetics to the technical corks; however, the oxygen desorption after bottling is higher and seems to take a longer time to reach the steady state. After this period, the OTR is relatively low, essentially occurring through the cork–glass interface, mainly after the first year of storage [17,40]. The mechanics that govern the oxygen diffusion through the cork structure remain unclear; however, some authors claim that oxygen crosses cell walls through the plasmodesmata channels, following Knudsen’s mechanics [41,42]. In contrast, other authors consider that gas transfer occurring inside the cork is possibly regulated by surface and/or molecular diffusions [34,36]. The oxygen ingress allowed by natural cork can vary according to several parameters related with its intrinsic properties, such as length, diameter, grade (coefficient of porosity), surface treatment, and cellar aging conditions such as position (vertical or horizontal), temperature, and relative humidity [17,43,44,45].

Several researchers have investigated the OTR of cork, synthetic, and screw cap closures in order to assess their role in oxygen entrance as well as their impact on the chemical and sensory characteristics of wines during bottle aging [14,29,46]. The effect of the oxygen barrier properties of closures on the aromatic composition, color, and sensory properties of a Bordeaux Sauvignon Blanc wine were investigated during 24 months of storage [46]. Results showed that wines sealed with a synthetic closure with the highest OTR had relatively *oxidized* attributes, a brown color, and lower levels of antioxidants (sulfur dioxide (SO_2_) and ascorbic acid) and volatile compounds (3-mercaptohexan-1-ol and hydrogen sulfide (H_2_S)) compared to wines sealed with other types of closures. On the other hand, wines sealed with Saran-tin screw caps with the lowest OTR had the slowest rate of browning, the highest levels of antioxidants and varietal thiols, as well as the highest levels of H_2_S, which were responsible for the *reduced* attributes found in these wines. Finally, wines sealed with cork stoppers (natural, agglomerated, and microagglomerated) and another type of screw cap (Saranex), all with intermediate OTR values, presented insignificant *reduced* and *oxidized* characteristics.

After 36 months of storage, Pinot Noir and Chardonnay wines sealed with natural cork, synthetic closures, and three different types of screw caps (Saran-tin, Saranex, and LDPE) revealed the highest OTR and lowest free SO_2_ and total SO_2_ in both wines sealed with LDPE screw caps, while the Saran-tin screw cap showed the lowest OTR and the highest free and total SO_2_ [29]. In addition, the concentrations of H_2_S, methanethiol, and thioacetates, which showed a decreasing behavior with bottle aging, were found at lower levels in LDPE screw caps and synthetic closures when compared with other closures. Higher levels of acetaldehyde were also found in both wines sealed with LDPE screw caps, highlighting the fact that higher OTR values could result in rapid oxidation of wine during bottle aging. Finally, the other two volatile compounds showed altered levels among the analyzed closures, namely lower concentrations of linalool and higher concentrations of *β*-damascenone in LDPE screw caps, both possibly associated with the oxidation process. 

The effect of natural cork, technical cork, and three types of synthetic closures on the flavor composition and sensory properties of Chardonnay wine after 48 months of storage was also studied by Liu et al. [14]. The results revealed higher free and total SO_2_ levels in wine sealed with natural and technical cork compared with synthetic closures. Bottle closures also had a significant impact on the levels of seven volatile compounds, namely acetoin, 1-butanol, 2-phenylethanol, 1-pentanol, (*Z*)-3-hexen-1-ol, 2-nonanol, and ethyl decanoate. Sensory analysis unveiled that cork closures, both natural and technical, and the two synthetic closures with the lowest OTRs preserved more *fruity* and *flowery* attributes, while the synthetic closures with the highest OTRs contributed more *grilled* characteristics to the wines.

Overall, these studies revealed three main tendencies of oxygen barrier properties of closures and their link with wine chemical composition and sensory attributes: (1) screw caps usually have lowest OTRs and the highest levels of antioxidants (SO_2_ and ascorbic acid) in the wine and are more susceptible to develop unpleasant *reductive* characters due to high levels of H_2_S; (2) synthetic closures have the highest OTRs and lowest levels of antioxidants in the wine and are usually associated with *oxidized* aromas and brown color in white wines; (3) cork stoppers have a wide range of OTRs, positioned between screw cap (lowest) and synthetic closures (highest), and an intermediate level of antioxidants in wine, preserving more *fruity* and *flowery* attributes. Other publications have corroborated these findings [47,48,49]. The impact of several closures on wine post-bottling development was also critically reviewed by Silva et al. [3], with special emphasis on oxygen exposure after wine bottling.

### 3.2. Desorption of Volatile Compounds from Closures into Wine

Several studies have reported that wine and wine model solutions can desorb several volatile compounds from cork (Table 2). Compound classes include alcohols, aldehydes, aromatic hydrocarbons, pyrazines, dicarbonyls, ketones, acids, furans, esters, monoterpenes, and sesquiterpenes. Aldehydes, ketones, and terpenes were the most representative compound classes, with 22 aldehydes, 15 ketones, 27 monoterpenes, and 5 sesquiterpenes identified in a total of 113 compounds. Aldehydes and ketones have been associated with the oxidative degradation of fatty acids present in wax and suberin fractions of cork [50] and are usually associated with unpleasant flavors [51]. Terpenes are produced by plants and are responsible for pleasant aromas, such as *sweet*, *herbal*, *citrus,* and *woody* [52,53]. According to Moreira et al., the terpenes that desorbed in the highest amount from cork granules into wine model solutions are L-camphor (234–418 ng/g) and *α*-terpineol (60–103 ng/L) [52]. Culleré et al. reported the sensory descriptive analysis and volatile composition of cork macerates, revealing that the presence of pleasant notes of *sweet/matured fruit*, *alcoholic*, *toasted*, *sweet wood,* and *flowery/muscat* in wine model solutions was correlated with the presence of esters, volatile phenolic compounds, and terpenes, among others [53].

Cork stoppers have also been described as capable of transmitting off-flavors to wine, namely TCA (*moldy* and *musty*), geosmin (*earthy*), 2-methylisoborneol (*musty/muddy*), 3,5-dimethyl-2-methoxypyrazine (*wet cardboard*, *musty,* and *dusty*), 3-isopropyl-2-methoxypyrazine (IPMP) (*green bell pepper*), and 3-isobutyl-2-methoxypyrazine (IBMP) (*vegetative* and *green*) [54,55,56,57,58]. According to several authors, the TCA content of cork that is desorbed into wine varies, on average, between 0.7% and 8% [54,55,56,57]. However, the cork industry was able to address this problem by implementing several technologies to remove taint compounds such as TCA from cork. These technologies include steam cleaning and thermal desorption processes as well as supercritical carbon dioxide extraction, among others. Recently, industrial gas chromatography technologies for individual screening, such as NDtech, have also been implemented to screen each natural cork for TCA and identified contaminated corks, resulting in natural corks completely free of TCA. Moreover, *trans*-4-*tert*-butylcyclohexanol was recently detected in a white wine sealed with a particular type of microagglomerated cork [49], possibly due to the composition of the binder or plastics used in the formulation of this type of closure.

Regarding synthetic closures, a desorption of monomers or additives from the polymer into the wine has been reported, leading to off-flavor generation and safety issues [67,68]. Plastic polymers suffer an incomplete polymerization during their synthesis, which leads to the presence of residual monomers or oligomers in the final product, which can be desorbed into the foods or beverages [69]. The desorption capacity depends on the chemical nature of the monomers or additives (e.g., volatility and polarity), the lipophilicity of the food matrix, the period of contact, and the temperature of storage [67]. Depending on the source of PE, different flavors can be developed, such as *candle*, *stuffy*, *musty*, *soapy,* and *rancid* [67]. Culleré et al. also reported the impact of maceration of synthetic closures in a wine model solution, unveiling the presence of unpleasant *rubber* and *mushroom* characters attributed to *m*-cresol and 1-hepten-3-one, respectively [53]. In addition, 2,4-Di-*tert*-butylphenol was identified in white wine and wine model solution sealed with synthetic closures after 48 months of storage [49]. This compound has been used in the plastic industry and the manufacturing of pharmaceuticals and fragrances for preparation of antioxidants and ultraviolet stabilizers [70], and its potential impact on wine sensory properties is still unknown. Recently, the presence of microplastics in several white wines sealed with synthetic closures was also reported [71].

### 3.3. Scalping of Volatile Compounds Present in Wine by Closures

The scalping phenomenon is characterized by the direct sorption of volatile compounds and other food constituents by the package materials [72,73]. Cork stoppers have been reported as capable of adsorbing compounds from wine; this can have a negative or positive impact on wine flavor [74,75]. However, this phenomenon has been more evident in synthetic closures, and it has not been reported in screw caps [75,76,77].

Regarding cork stoppers, researchers have found that although wine can desorb a small proportion of TCA from this type of closure, a much higher proportion of TCA and other chloroanisoles is adsorbed by natural and agglomerated cork stoppers from wine [74,76]. Natural and technical cork stoppers also have the ability to partially adsorb ethyl octanoate and ethyl decanoate, with an increased capacity of adsorption proportional to the increased ester chain length [76]. Naphthalene has also been reported as adsorbed by natural and technical corks [76]. TDN was described as the most strongly affected by natural and technical cork stoppers [47,76,78,79]. This molecule, although unpleasant in other wine cultivars, is characteristic of Riesling aged white wines, conferring a particular *kerosene* flavor [80]. Volatile phenolic compounds, such as guaiacol, 4-methylguaiacol, 4-ethylguaiacol, 4-propylguaiacol, 4-vinylguaiacol, 4-ethylphenol, and eugenol, which are commonly present in wine and are responsible for conferring negative attributes, can also be adsorbed from wine by natural cork via weak interactions with the cork surface [66]. Interestingly, the capacity of suberin to sorb volatile phenols has been proven, unveiling a high sorption capacity positively correlated to the hydrophobicity of the volatile compounds [81]. Finally, natural and agglomerated cork stoppers have shown adsorptive capacity for methoxypyrazines, namely IBMP, IPMP, and 3-*sec*-butyl-2-methoxypyrazine (SBMP) [82]. These compounds are potent odor-active constituents of wine and are responsible for masking the *fruity* and *floral* aromas and generating undesired attributes similar to *green bell pepper* and *vegetable*.

Synthetic closures have shown a much higher capacity to adsorb non-polar compounds than cork stoppers due to the polyolefinic nature of PE, which confers high lipophilicity [67,83]. Thus, this polymer can adsorb volatile compounds, organic acids, and pigments from the wine, which could lead to the loss of aroma intensity and fruitiness, resulting in the development of unbalanced wine flavor characteristics [67,73]. PE film has shown an effective adsorption of chloroanisoles from wine [74], with the concentration of all chloroanisoles reaching an equilibrium within three days of contact of wine with PE film at room temperature. Furthermore, PE film in contact with wine samples for four days was responsible for decreasing its *floral* and *fruity* aromas [74]. In comparison with natural and technical cork stoppers, synthetic closures showed a significantly greater adsorption of esters (ethyl hexanoate, ethyl octanoate, and ethyl decanoate), naphthalene, and TDN [76]. In addition, the monoterpene rose oxide, which gives a *lychee* character to some white wines, was partially adsorbed only by the synthetic closures. This type of closure also showed a greater capacity for adsorption of methoxypyrazines (IBMP, SBMP, and IPMP) when compared with natural and agglomerated cork stoppers [82].

Overall, these studies clearly indicate that the presence of synthetic materials in the closures, which is in direct contact with bottled wines, is detrimental to their quality as a result of the scalping of several volatile compounds, such as esters and organic acids, leading to aroma intensity and quality losses.

## 4. Conclusions

This review shows that wine matrix composition, bottling conditions, and/or closure barrier properties have a significant impact on the sensory quality of wines. Winemaking does not end at bottling; it continues on into the post-bottling phase. Therefore, the packaging choice is the last and one of the most important decisions of the winemaker; it directly impacts the sensory quality of bottled wine presented to consumers. For this decision, it is crucial to understand the different properties of closures, namely the oxygen ingress and OTR and the desorption/scalping behaviors under real conditions of use. Synthetic closures exhibit some properties that seem to be detrimental to wine flavor quality: high OTR, leading to the development of oxidative aromas in wine, and very high scalping capacity, which reduces the fruitiness and flowery characters of wine and its aroma complexity. In addition, several compounds as well as nanoplastic and microplastic particles can also be leached into the wine; it remains unclear how these compounds affect the sensory and food safety properties of wines. In contrast, screw cap closures present the lowest OTR and the lowest scalping capacity, preserving the fruitiness and freshness, leading bottled wine towards a more reductive development. The literature also suggests that screw caps can leach some metals into wines, but there is limited evidence on how this phenomenon can impact bottled wines.

Finally, cork stoppers exhibit OTR values lower than synthetic closures but higher than screw caps. Among cork stoppers, technical corks such as agglomerated and microagglomerated present lower OTRs, similar to screw caps; however, the oxygen ingress is higher given the oxygen desorption phenomenon. Natural cork stoppers diffuse out more oxygen than the technical corks, allowing some controlled wine micro-oxygenation during bottle aging. Cork can preserve the aroma of wines, as it is a material with low scalping capacity towards certain non-polar compounds responsible for wine fruitiness. Notwithstanding, cork can transfer tiny amounts of its own compounds to wine, which, by their reaction with wine compounds, can be beneficial to wine’s aroma flavor complexity (e.g., phenolics, ethyl esters, and terpenes). However, the importance of this cork contribution for wine organoleptic properties during bottle aging remains unclear. Therefore, more studies are needed to better understand cork/wine interactions in order to define the best fit that will maximize wine quality and optimize shelf life.

## Figures and Tables

**Table 1:** General overview of oxygen transmission rate (OTR) values (mg/closure/month) obtained or recalculated from the literature for the main wine closures.

Closures		Colorimetric *	Chemiluminescence	Coulometric
Cork stoppers	Technical	0.003 to 0.004	0.025 ^*e*^ 0.050 ^*e*^	0.03 to 0.05 ^*b*^
	Natural	0.007 to 0.010	NA	0.004 to 5.0 ^*b*^ 0.001 to 0.051 ^*d*^
Synthetic	Co-extruded	0.033 0.065	0.050 ^*f*^ 0.091 *^f^* 0.141 *^f^*	0.040 ^*b*^ 0.17 to 0.31 *^d^* 0.7 to 1.4 *^e^*
Screw caps	Saran-tin	0.001	0.010 to 0.012 *^c^*	<0.001 *^a^* 0.008 ^*b*^ 0.003 to 0.0039 *^c^*
	Saranex	0.02 to 0.03	0.045 to 0.065 *^c^*	0.03 *^b^* 0.02 to 0.03 *^c^*

* Calculated after steady state reached (6 months). *^a^*^, *b*, *c*, *d*, *e*, *f*^ According to [25], [26], [27], [28], [29], [30], respectively. NA—not analyzed.

**Table 2:** List of volatile organic compounds reported to be desorbed from cork into wine or wine model solution.

Compound	CAS	Odor Descriptor	References
*Alcohols*			
3-Methyl-1-butanol	123-51-3	*Whiskey ^a^*	[59]
1-Octen-3-ol	3391-86-4	*Mushrooms ^a^*	[59]
1-Octanol	111-87-5	*Wax ^a^*	[59]
Benzyl alcohol		*Roses, almond*	[60]
Geosmin	19700-21-1	*Earth, musty*	[58,59]
Isobutanol	78-83-1	*Flowery, anise*	[53]
Phenylethyl alcohol	60-12-8	*Flowers, honey*	[60]
*Aldehydes*			
Propanal	123-38-6	*Fruity, fresh green ^b^*	[52]
Butanal	123-72-8	*Fruity, burnt, sweet ^b^*	[52]
Pentanal	110-62-3	*Dry fruit, nutty*	[52]
Hexanal	66-25-1	*Grass, herbaceous ^b^*	[52,61]
Heptanal	111-71-7	*Fatty, rancid ^b^*	[52,61]
Octanal	124-13-0	*Lemon*	[52,53,61]
Nonanal	124-19-6	*Herbal, citrus ^b^*	[52,61]
Decanal	112-31-2	*Fruity, citrus ^b^*	[52,61]
Undecanal	112-44-7	*Citrus, floral ^c^*	[52]
2-Propenal	107-02-8	*Almond, cherry ^c^*	[52]
(*E*)-2-Butenal	123-73-9	*Flower ^c^*	[52]
(*E*)-2-Pentenal	1576-87-0	*Fruity, green ^c^*	[52]
(*E*)-2-Hexenal	6728-26-3	*Almond, fruity ^c^*	[52]
(*E*)-2-Heptenal	18829-55-5	*Fatty, green ^c^*	[52]
(*E*)-2-Octenal	2548-87-0	*Fatty, herbal ^c^*	[52]
(*E*)-2-Nonenal	18829-56-6	*Green, cucumber ^c^*	[52,60]
(*E*)-2-Decenal	3913-81-3	*Fatty, oily ^c^*	[52]
2-Methyl-1-propanal	78-84-2	*Fruity, malty ^b^*	[52]
2-Methyl-1-butanal	96-17-3	*Almond, nutty ^c^*	[52]
3-Methyl-1-butanal	590-86-3	*Fruity, cheesy ^b^*	[52]
Benzaldehyde	100-52-7	*Bitter almonds ^b^*	[52,61]
Phenylacetaldehyde	122-78-1	*Floral, honey ^b^*	[52]
*Benzenoids*			
*o*-Cymene	527-84-4	-	[61]
Naphthalene	91-20-3	*Pungent, tarry ^c^*	[61]
Guaiacol	90-05-1	*Phenolic, spicy*	[53,59,62]
4-Vinylguaiacol	7786-61-0	*Wood, spice, curry*	[60]
Methyl guaiacol	91-16-7	*Leather, spicy ^d^*	[62]
Eugenol	97-53-0	*Spice, cloves, honey*	[60]
Isoeugenol	97-54-1	*Carmination*	[60]
Cerulignol	2785-87-7	*Spicy*	[60]
2,4,6-Trichloroanisole (TCA)	87-40-1	*Musty, earthy, moldy*	[54,56,58,59,62,63]
*m*-Cresol	108-39-4	*Leather*	[53]
Vanillin	121-33-5	*Vanillin*	[53,60]
Methyl vanillate	3943-74-6	*Vanillin*	[53]
*Pyrazines*			
MDMP	-	*Musty, dusty*	[58]
IPMP	25773-40-4	*Green, vegetative*	[58]
IBMP	24683-00-9	*Green bell pepper*	[58]
*Dicarbonyls*			
Diacetyl	431-03-8	*Buttery, cream*	[52,53]
Glyoxal	107-22-2	-	[52]
Methylglyoxal	78-98-8	-	[52]
*Ketones*			
Propan-2-one	67-64-1	*Apple, ethereal ^c^*	[52]
2-Butanone	78-93-3	*Fruity, acetone ^b^*	[52]
3-Methyl-2-butanone	563-80-4	*Camphor ^c^*	[52]
2-Pentanone	107-87-9	*Fruity ^b^*	[52]
2-Hexanone	591-78-6	*Ether ^c^*	[52]
2-Heptanone	110-43-0	*Fruity, herbal ^c^*	[52]
3-Penten-2-one	625-33-2	*Fishy, phenolic ^c^*	[52]
4-Heptanone	123-19-3	*Fruity, sweet ^c^*	[52]
2-Cyclohexen-1-one	930-68-7	*Green, roasted ^c^*	[52]
6-Methyl-5-heptanone	13019-20-0	*Fruity, green ^c^*	[52]
2-Octanone	111-13-7	*Bitter, earthy ^c^*	[52]
2-Nonanone	821-55-6	*Fresh, herbal ^c^*	[52]
2-Decanone	693-54-9	*Fatty, floral ^c^*	[52]
2-Undecanone	112-12-9	*Fresh, floral ^c^*	[52]
1-Octen-3-one	4312-99-6	*Mushroom*	[53]
*Acids*			
Octanoic acid	124-07-2	*Coconut, rancid, cheese*	[60]
Vanillic acid	121-34-6	*Vanilla*	[60]
Nonanoic acid	112-05-0	*Wax, dry, fatty*	[60]
Dodecanoic acid	143-07-7	*Coconut, fatty, metallic*	[60]
Benzeneacetic acid	103-82-2	*Honey, fruity, sour*	[60]
*Furans*			
Furfural	98-01-1	*Toasty, caramel ^b^*	[52,60]
5-Methyl-2-furfural	620-02-0	*Spicy, toasty ^b^*	[52]
*Esters*			
Ethyl hexanoate	123-66-0	*Fruity, brandy ^b^*	[61]
Ethyl heptanoate	106-30-9	*Fruity, nutty ^b^*	[61]
Ethyl nonanoate	123-29-5	*Fruity, waxy ^c^*	[61]
Fenchyl acetate	13851-11-1	*Citrus, herbal ^c^*	[61]
Isobornyl acetate	125-12-2	*Herbal, woody ^c^*	[61]
Ethyl isobutyrate	97-62-1	*Fruity, strawberry*	[53]
Ethyl 2-methylbutyrate	7452-79-1	*Fruity, green apple*	[53]
Ethyl isovalerate	108-64-5	*Fruity, anise*	[53]
3-Methylbutyl acetate	123-92-2	*Fruity, anise*	[53]
Ethyl butyrate	105-54-4	*Fruity*	[53]
Butyl acetate	123-86-4	*Grass*	[53]
*Monoterpenes*			
*α*-Pinene	80-56-8	*Minty ^c^*	[52,61]
Camphene	79-92-5	*Herbal, woody ^c^*	[61]
*β*-Pinene	80-56-8	*Green, hay ^c^*	[61]
1,4-Cineole	470-67-7	*Minty, pine ^c^*	[52,61]
Citronellol	106-22-9	*Citrus, floral ^c^*	[62]
*α*-Terpinene	99-86-5	*Citrus, herbal ^c^*	[52,61]
Limonene	5989-54-8	*Lemon, orange ^c^*	[52,61,62]
Eucalyptol	470-82-6	*Mint, herbal ^c^*	[52,61]
Terpinolene	586-62-9	*Pine, woody ^c^*	[61,62]
Fenchone	1195-79-5	*Earthy, herbal ^c^*	[52,61]
Fenchol	1632-73-1	*Lemon, pine ^c^*	[52,61,62]
*α*-Campholenal	4501-58-1	*Green, leafy ^c^*	[61]
L-Camphor	464-49-3	*Camphor ^c^*	[52,60,61,62]
*trans*-*β*-Terpineol	7299-40-3	-	[61]
*trans*-3-Pinanone	547-60-4	*Spicy ^c^*	[61]
Isoborneol	124-76-5	*Herbal, woody ^c^*	[52,61]
2-Methylisoborneol	2371-42-8	*Musty, muddy*	[58]
L-Borneol	464-45-9	*Camphor, anise*	[52,53,61,62]
2-Methylisoborneol	2371-42-8	*Earth, musty ^a^*	[59]
*cis*-3-Pinanone	15358-88-0	*Camphoreous, cedar ^c^*	[52,61]
*α*-Terpineol	98-55-5	*Floral, mint ^c^*	[52,53,61]
1-Terpineol	7785-53-7	*Floral, lilac ^c^*	[52]
4-Terpineol	562-74-3	*Earth, musty ^c^*	[52,61]
Linalool	78-70-6	*Flowery, muscat*	[53,62]
*cis*-Linalool oxide	11063-77-7	*Earthy, sweet ^c^*	[52]
L-(-)-Menthol	2216-51-5	*Minty, peppermint ^c^*	[52]
2-Pinen-4-one	18309-32-5	*Menthol ^c^*	[52]
*Sesquiterpenes*			
*α*-Copaene	3856-25-5	*Spice, woody ^c^*	[61]
D-Longifolene	475-20-7	*Rose, sweet ^c^*	[61]
*β*-Cadinene	523-47-7	*Green, woody ^c^*	[61]
L-Calamenene	483-77-2	*Herb, spice ^c^*	[61]
Eremophila ketone	158930-41-7	-	[61]

*^a^*^, *b*, *c*, *d*^ According to [59], [64], [65] and [66], respectively. - Not available.
